# Observations on Select Subjects in Surgery

**Published:** 1803-07-01

**Authors:** W. Simmons

**Affiliations:** Manchester


Medical and Physical Journal.

VOL. X.] JULY 1, 1803. [NO. LIII.

Printed for R. PHILLIPS, by W. Thorne, Red Lion Court, Fleet Street, London.

### OBSERVATIONS *on* SELECT SUBJECTS *in* SURGERY; *communicated by* Mr. W. SIMMONS, *of Manchester*.

### ON A CASE OF ABSCESS OF THE LIVER.

INFLAMMATION of the Liver is not an uncommon distemper, and is frequently brought on by excess in the use of fermented liquors. As in other parts of the body, it is here obedient to the several terminations of inflammation; but the one by abscess is to be deprecated, as little less alarming than the original malady. I know not whether it be sufficiently in evidence at present, that, in the suppurative stage, a difference of result may be expected, as the exciting cause is external or internal, or as the abscess is situated upon the surface or in the substance of the viscus; but as the point must eventually be determined by the preponderance of facts, it may not be useless to record the one following.

A chair-maker, of middle age, was employed for nine months together in boring circular holes in the feet to receive the spindles, by which the fore and hind feet of a chair are fastened. In this occupation, the head of the brace or borer had pressed upon the pit of the stomach; and, at length, produced inflammation and suppuration in that portion of the liver lying under it. Of the early circumstances the man himself gave a particular and distinct account; and subsequently report and opinion of his physician, left no room to hesitate of the nature of the case. The enlargement of the belly was chiefly confined to the right side, though latterly it had spread a good way, and in every part a deep seated fluctuation was distinctly perceptible.

Lest a spontaneous rupture might occur, and the matter escape into the abdominal cavity, it was advised in consultation to make an opening without delay. The parietes did not any where protuberate, and in making it, therefore,

I proposed to approach the seat of the complaint. With this intention, the incision was begun at the pointed extremity of the cartilago ensiformis, and continued downward in the line of the navel to the extent of four inches. An aperture was next made into the containing cyst, and a blunt pointed bistoury introduced, to enlarge it as quickly as possible to the capacity of the external wound. The matter, neither thin nor discoloured, then streamed out very copiously; not less than a gallon of which was caught in a large wash-hand bason, and at least two quarts more were spilt upon the floor of the operation room. He was greatly relieved by the discharge, but as the small lobe of the liver lay conspicuous at the bottom of the wound, the expectations of recovery were very moderate. Light dressings however were applied, and the lips, brought almost in contact, were kept from receding by means of adhesive plaster; and, besides these, the customary precautions after a capital operation were duly regarded. Whatever apprehensions of danger might be justly formed, the consequent symptoms were at no time alarming, and in two months, the discharge of matter having ceased, the wound was permitted to close. Two years afterwards, I had occasion to know that the cure was permanent; nor, in the site of the incision, was there any disposition to hernia. The exposed portion of the liver granulated in a manner more beautiful than I ever saw in any other part of the body.

### On the Incontraction of an Artery.

In his late work on the diseases of the knee-joint, Mr. Russell has described a complaint which he has been pleased to call, "an uncommon disease," and Mr. Hey has since published many cases of it under the title of "Fungus hæmatodes." Hitherto my own experience has been very limited, for I have seen only two cases; but as those have impressed me with an opinion of the nature of the distemper different from either of the above gentlemen, it may not be altogether useless to relate it. The disease is however less novel than had been imagined, as, in his Remarks on Fractures, Mr. Pott had described it, and assigned a *bursten* artery for the cause. Of those two cases, which I have seen, one only fell under my own management, and in that amputation was performed with success. It occurred in a young gentleman, who ascribed it to an over exertion in jumping, as he was sensible of something giving way at the time in his right leg, and, soon after, it began to tumefy in that part. This happened eighteen months months before I saw him, during which the swelling had progressively augmented in bulk. At that time, it extended from just below the knee to a little above the ankle, and bulged forward considerably; but, though resisting, neither fluctuation nor pulsation could be distinguished in it. From this account it will appear, that there was a good deal of obscurity about it, which it was my first object to remove. To this end the cavity was laid open by a longitudinal incision, which gave vent to a prodigious collection of a gelatinous substance, and exposed the bottom and sides, of which the interosseous ligament constituted the former; and the inner surface of the tibia and fibula, denuded of periosteum, served for the latter. But nothing like muscle could be found, the whole forming one homogeneous mass. As an opinion had arisen that the disease was an aneurism, the anterior tibial artery was sought for, but the trunk of it was destroyed, leaving only just enough after it had passed through the ligament to apply a ligature. This, and several others, the orifices of which were open, were secured in that manner, and great hopes were then entertained of saving the limb. But, on removing the tourniquet, the effusion of blood from the general surface was so great, as to threaten with immediate danger, which, together with the probability of extensive exfoliations of bone, and of a long and exhausting discharge, rendered it expedient to have recourse forthwith to amputation, which was accordingly performed.

My opinion of this malady at that time, and better information has only served to confirm me in it, that by jumping the anterior tibial artery was ruptured, and never afterwards perfectly closed. Any slight exertion would therefore open it again, and occasion a fresh effusion of blood. Nor is this at all extraordinary, for we know that it is sometimes necessary, long after an amputation performed even in consequence of an accident, to apply ligatures still higher up upon the trunk of the artery to stay a more recent hæmorrhage, and sometimes even to repeat it. In one instance indeed the patient was nearly exhausted by its frequent recurrence, and the propensity to hæmorrhage could only be corrected by a diet of milk and vegetables; and several I have known sink under it. From the blood thus effused, the serous portion and red particles are absorbed, leaving the coagulable lymph behind to form the contents of the sac. But as the growth of the tumour, as in the above case, may be slow, it is probable that the quantity of extravasation is limited at each time,

 not not only by the degree of impelling force, but contrariwise by the incumbent weight and exterior resistance, causes which in their operation would also gradually dwindle both by a partial absorption and an enlargement of the parietes.

The next symptom recorded by Mr. Russell and Mr. Hey that I shall attempt to explain, is a caries of the adjacent bone or bones, an effect which we know may be produced by pressure, even supposing the extravasated lymph not to possess a dissolvent property. However, from the rapidity of the process in general, I should be disposed to favour this opinion; for, although in my own case the bones were merely bare, in the other that I saw, the boney structure within the sphere of its agency was almost entirely destroyed in about nine months.

The issue of arterial blood, which is observable upon parting the masses of viscosity, has led to an opinion that they have an organized structure, but I think erroneously; for, if we suppose the blood to have been recently effused, without undergoing any change, and only to have found a vent by the separation, how easily is that phenomenon resolved? I certainly saw nothing like organization in either case that fell under my observation; and, from every consideration of the subject, I cannot yield assent to the existence of it, until injections have fully established the fact.

The last symptom of which I shall offer an explanation, is the absence of all pulsation in the tumour. And, if we consider the undilated state of the artery, and the thickness of the substances interposed between it and the hand, it can hardly be expected that its motions should be perceived. Indeed, it is rather probable that the sides of its opened extremities are brought nearly together by the causes before mentioned, which would diminish the force of its action correspondently.

In the advanced stage of this complaint, our only resource is in amputation; and even this may fail, if the whole of the arterial trunk is morbidly altered. But, when the cause is external, the early securing of the artery before much mischief is done to the contiguous parts, may be the means of preserving the limb, and give as effectual security against the recurrence of hæmorrhagy, as the tying of the artery after amputation.

### On an Incarcerated Hernia during Pregnancy.

A lady, who had been long tormented both with an umbilical and femoral hernia, the latter of which had been often often strangulated and reduced; in the year 1792, experienced a fresh descent, which proving intractable to the usual means, my assistance was requested. For two whole days every exertion was made to return it, but without success, so that on the morning of the third, she submitted to the operation. About six inches of the ileum were protruded, and a good deal discoloured; however, soon after the replacement, she grew tolerably easy, and in a little while had a motion, which afforded much relief. Afterwards, the symptoms gradually abated, and the wound uniting by the first intention, she was confined only for a few weeks. The only thing remarkable in the herniary sac was the convoluting upwards of the intestine, in consequence of which the portion first protruded was opposed to the efforts by the taxis, and consequently the reduction could not be accomplished in that way. But, during her recovery, there was reason to believe that she was two months gone with child at the time of the operation; and, seven months from that juncture, I delivered her of a fine healthy girl, after an easy and natural labour.

That during the incarcerated state of the intestine, the almost incessant vomiting, the attempts at reduction in every variety of posture, the continuance of cold applications and even ice to the tumour, the clysma fumosum, and other means, and lastly, the operation itself, should not have produced abortion, is indeed extraordinary, especially when we consider how trifling is the cause of it in many instances, and, when once formed, how difficult is the habit to be conquered.

### On a Wound of the Trachea.

While some practitioners have looked upon a wound of the trachea as being of little moment; others have regarded it as certainly mortal. Probably both those opinions are in the extreme; because many recover after such an accident, though it is never to be lightly treated. When the inflammation spreads from the wound down the trachea, it produces an extreme degree of danger, a source of it that has not I think been sufficiently insisted upon, on which account I shall subjoin a case that may furnish an instructive example.

A respectable young woman had nearly divided the trachea with a very sharp knife, in a paroxysm of insanity; and, although neither of the carotids was wounded, she lost a good deal of blood. Assistance being at hand, the borders of the external wound were held in contact by a couple of sutures, till I could see her a few hours afterwards, when she was perfectly sensible, and her mind was tolerably composed. Her head was now inclined forward upon the chest as far as it conveniently could be, and secured in that position by proper bandages. Another suture was also added, more effectually to close the external wound, and over all was given the additional security of adhesive plasters. No symptom of alarm occurred during the first three days; but, early on the fourth, she was seized with the croup. I saw her in the afternoon, and immediately bled her *ad deliquium,* and as soon as she was a little recovered, half a score leeches were applied to the nape of the neck. The symptoms were nevertheless urgent next morning, and, as the pulse would bear it, I bled her again to the former effect. A large blister likewise was laid between the shoulders after the first bleeding, and another upon the sternum after the second. The bowels also were freely emptied by calomel; and fomentations to the feet, and other auxiliary means, were duly persisted in. However, on the third day from this seizure, there did not appear to be the smallest chance of her recovery; but, on the fourth, she began to expectorate; and thence forward the symptoms gradually subsided, and in three weeks the wound was completely healed.

I never saw a case of croup more distinctly marked than this, and it yielded to a similar method of treatment, except in the use of emetics, which, for reasons too obvious to need mentioning, could not here be given.

Although Bronchotomy is an operation recommended on several occasions, yet I have known no instance of the performance of it on the living subject. In the advanced stage of the croup, where benefit has been expected, in my opinion, no solid advantage can be derived. But as the objections which I have to offer are not new, and are so well set forth in Dr. Friend's History of Physic, Vol. I. p. 203, Ed. 3, I trust it will not prove unacceptable to read the passage in this place. It stands thus: "We see how conversant *Paulus* was in the most difficult operations of surgery; and as he seems to understand the nature of the cases he treats of, so we shall find him no less well acquainted with the manner of curing them. I must observe further, that there are some operations he gives an account of, which are neither described nor recommended by any author now extant before him, one of which is *bronchotomy,* or opening the wind-pipe wind-pipe in a violent *quinsy*. The manner of this process he takes from Antyllus, which, because it is new and exact, give me leave to transcribe here. Our best surgeons have described this operation; Antyllus particularly thus: We think this practice useless, and not to be attempted where all the arteries (I suppose he means all the branches of the aspera arteria) and the lungs are affected; but when the inflammation is chiefly about the throat, the chin, and the tonsils which cover the top of the wind-pipe, and the *artery* is unaffected, this experiment is very rational to prevent the danger of suffocation. When we proceed to perform it, we must cut through some part of the wind-pipe, below the larynx, about the third or fourth ring; for to cut quite through would be dangerous. This place is the most commodious, because it is not covered with any flesh, and because it has no vessels near it. Therefore bending the head of the patient backwards, so that the wind-pipe may come more forward to view, we make a transverse section *between two of the rings*, so that in this case not the *cartilage* but the *membrana*, which encloses and unites the cartilages together, is divided. If the operator be a little fearful, he may first divide the skin extended by a hook, then proceeding to the pipe, and separating the vessels if any are in the way, he must make the incision. Thus far Antyllus. Paulus adds, that he (Antyllus) thought upon this way of cutting, by observing, (when it was, I suppose, cut by chance) that the air rushed through with great violence, and that the voice was interrupted. When the danger of suffocation is over, the lips of the wound must be united by suturæ, (i. e.) by sewing the *skin* and not the *cartilage*; then proper vulnerary medicines to be applied. If these do not agglutinate, an incarnant must be used. The same method must be pursued with those who cut their throats with a design of murdering themselves."

"The operation we see is very clearly described, and some observations extremely proper are made upon it. C. Aurelianus ridicules this operation as fabulous, and as never practised by any of the ancients, and says it is only a rash invention of Asclepiades. *Aretæus* mentions it too, but thinks it not warranted by experience; that the wound would endanger an inflammation, cough, and strangling; and if the danger of being choaked could be avoided by this method, yet the parts would not heal, as being cartilaginous! But *Paulus*, I think, answers these objections; and it is certain that some of the moderns have attempted this practice with success: however, it is reckoned a dangerous undertaking." I shall add only one observation to this long extract, which is, that, if a simple incised wound could produce so violent an inflammation of the trachea, as the case of my patient has evinced, what must be the consequence of keeping a silver canula in the opening for several days together, after the operation of Bronchotomy?

I should now have concluded, but for a remark concerning union by the first intention after an operation contained in the next page but one of the same volume, that had escaped my recollection, although it is not quite relevant to the prefent enquiry. Still speaking of Paulus, whom Dr. Friend places in the year 620, he further observes: " An operation, never described before, is taking off the breasts in men, when they grow, as they sometimes do, to any immoderate bigness. In this case, a good deal of fat, he says, grows underneath, and resembles a woman's breast; and therefore ought to be removed by the hand of the surgeon. The process runs thus: " A lunar section must be made in the lower part of the breast, and after the fat is taken out the skin is to be sewed together. If it is very prominent, and hangs over as in women, there must be two lunar sections made, meeting one another at their extremities; and when the fat and skin are removed, the wound is to be sewed up; and if any thing be left behind, the operation is to be repeated a third time."

So much for union by the first intention, after a chirurgical operation, as a modern discovery.

### On a Case of Ulcerated Leg.

In expressing my sentiments on the utility of the plaster-bandage in the treatment of ulcers, which are contained in a former number of the Medical and Physical Journal, my aim was to furnish the practitioner, whose opportunities of trying it are more limited than my own, with such practical intelligence as would conduct him to its successful employment. I have now to offer a solitary case, in order to impress several points on the reader's attention more strongly than before.

In the month of April last, a middle-aged single woman put herself under my care for ulcers upon her right leg of fourteen years standing, the condition of which may be in part conjectured by knowing that she had never obtained any regular assistance for them, but had contented herself with such applications as herself or her friends could could suggest. The whole of the limb was nearly covered with ulcers, but the larger ones were situated above the inner and outer ancle, and occupied a space almost entirely around it. The lips of those two in particular were callous and inverted, and their surfaces, which were foul, yielded profusely a fœtid, sanious discharge. And, in addition to these, a deep-seated, indolent tumefaction pervaded the whole of the parts unulcerated. Her general health also was much impaired by the severity of the pain, the long and copious drain of fluids, and from the hopeless prospect before her; for she had long abandoned all thoughts of relief except from amputation. In this state of the case, the plaster-bandage was applied with care from the toes up to the knee, and over it were secured bolsters of cloths by means of a callico roller. She thought the pain rather increased by the first dressing, which was probably caused by the plasters not being drawn sufficiently tight, as, on persuading her not to meddle, she became easier after the second, and subsequently experienced little pain. From that time too the quality of the discharge began to ameliorate, and the deeply ulcerated excavations shewed a disposition to incarn; the callous borders were gradually flattened, and the general tumefaction subsided with haste. The skinning process then became active, and spread with celerity in the usual order from the circumference towards the centre, except in the inner large ulcer above described, which healed inversely though with not less rapidity, from the centre towards the circumference; and within the short space of a month, the whole was completely cicatrized.

This is the most striking example of the speedy cure of so inveterate an ulcer that I have met with, and it hath convincingly demonstrated to me that the production of new skin is wholly independent on any elongation from the border; for in that that healed from the centre, not less than the breadth of my hand had skinned over in a single night, and the new skin continued to expand until it came in contact with the original skin, with which it then conglutinated.

Let me now take occasion to observe, that since I have employed the adhesive bandage in this species of ulcer, the insertion of an issue towards the completion of the cure has been dispensed with. Nor have I observed any inconvenience to arise from the suppression of the discharge; indeed, it is probable from the sudden alteration for for the better produced by compression, that its condition depends most commonly at least on local causes.

### On a Case of early Cataract.

It has been generally understood among practitioners, that the cataract occurring in early infancy does not admit of an operation before the approach of manhood. So prevalent is this opinion, that few, I believe, have undertaken it at an earlier period. But, if it should appear hereafter that it may be executed with success before so mature an age, as the advantages of a more early education would be thereby obtained, no further argument can be required to invite the performance of it. It is with great pleasure, therefore, that I submit the following case to the public attention.

Thomas Worrall, of Lichfield, a delicate boy, nine years of age, was admitted into the Infirmary on Monday the fourth of April last, for a cataract in each eye, that deprived him of sight. They were first discerned at about six months, and, before the end of the year, both the pupils were obscured. As his general habit did not require any preparation, the cataract in the left eye was depressed on the Saturday following, and, on the third day, he could distinguish objects. Nevertheless, at a subsequent examination, I was mortified to find that the cataract had risen again. But, as the dislodgment of it was complete at the time of the operation, and it was so lacerated that even a portion had escaped before the iris, no further attempt was made, and before the second operation the pupil was become tolerably clear. On that day three weeks the cataract in the right eye was depressed, and with complete success. In both these operations, I employed the needle lately recommended by Mr. Hey, whose commendation of it is here fully justified by the event, for neither inflammation nor pain ensued; and the only medicines my patient took, were an anodyne and an aperient draught after the first operation.

I must candidly confess, however, that it has less power than the spear-pointed needle, and is also less fitted to entangle the capsule when adherent; but these objections were successfully combated in the second operation, by passing the instrument quite through the upper portion of the cataract.

*June*, 1803.

